# Nucleobindin 2 inhibits senescence in gastric carcinoma

**DOI:** 10.1038/s41598-024-61111-5

**Published:** 2024-05-17

**Authors:** Yu Ishibashi, Takashi Itoh, Yasuko Oguri, Miki Hashimura, Ako Yokoi, Toshihide Matsumoto, Yohei Harada, Naomi Fukagawa, Misato Hayashi, Mototsugu Ono, Chika Kusano, Makoto Saegusa

**Affiliations:** 1 Department of Pathology, Kitasato University School of Medicine, 1-15-1 Kitasato, Minami-ku, Sagamihara, Kanagawa 252-0374 Japan; 2 Department of Gastroenterology, Kitasato University School of Medicine, 1-15-1 Kitasato, Minami-ku, Sagamihara, Kanagawa 252-0374 Japan; 3 Department of Pathology, Kitasato University School of Allied Health Science, 1-15-1 Kitasato, Minami-ku, Sagamihara, Kanagawa 252-0374 Japan

**Keywords:** NUCB2, Senescence, Proliferation, Apoptosis, Gastric carcinoma, Cancer, Cell biology, Gastroenterology, Oncology

## Abstract

Here, we focused on the role of Nucleobindin 2 (NUCB2), a multifunctional protein, in gastric carcinoma (GC) progression. NUCB2 expression was investigated in 150 GC cases (20 non-invasive (pT1) and 130 invasive (pT2/pT3/pT4) tumors) by immunohistochemistry (IHC), and in situ hybridization for detection of the mRNA in 21 cases. Using GC cell lines, we determined whether NUCB2 expression was associated with specific cellular phenotypes. In GC clinical samples, NUCB2 was transcriptionally upregulated when compared to normal tissues. High NUCB2 expression was associated with clinicopathological factors including deep tumor invasion, lymphovascular invasion, lymph node metastasis, and advanced clinical stages, and was a significant independent predictor of unfavorable progression-free survival in 150 non-invasive and invasive GC patients. Similar findings were also evident in 72 invasive GC cases in which patients received post-operative chemotherapy, but not in 58 invasive tumors from patients who did not receive the chemotherapy. In cell lines, NUCB2 knockout inhibited proliferation, susceptibility to apoptosis, and migration capability by inducting cellular senescence; this was consistent with higher proliferation and apoptotic indices in the NUCB2 IHC-high compared to NUCB2 IHC-low GC cases. NUCB2-dependent inhibition of senescence in GC engenders aggressive tumor behavior by modulating proliferation, apoptosis, and migration.

## Introduction

Gastric carcinoma (GC) is a disease with a multifactorial etiology; diet, lifestyle and several environmental risk factors all contribute to onset of GC^1,2^. It is the fifth most common malignancy worldwide with approximately 1.1 million new cases that occur mainly in Asian (particularly China) and South American countries^3–5^. In addition, it is the fourth leading cause of cancer mortality, with around 800,000 deaths per year^3,4^. Although treatment with aggressive and adjuvant chemotherapy in advanced GC has improved survival rates, the prognosis remains poor due to carcinoma invasion and metastasis^6^. Therefore, identification of the molecular mechanism(s) underlying GC progression may further improve patient survival.

Nucleobindin 2 (NUCB2) is a precursor protein of nesfatin-1, which was ordinally identified in hypothalamic nuclei and associated with food intake and energy homeostasis^7,8^. The protein has characteristic functional domains including a Leu/Ile rich region, two Ca^2+^ binding EF-hand domains separated by an acidic amino acid-rich region, and a leucine zipper^9^. The role of NUCB2 in tumor development and metastasis is Janus-like. For example, high NUCB2 expression is associated with cell migration and shorter recurrence-free survival time in prostate, clear renal cell, and breast carcinomas^10–12^, whereas NUCB2 and nesfatin-1 inhibit proliferation in adrenocortical and ovarian epithelial carcinomas^13,14^.

Cellular senescence is a dynamic, multistep program that results in permanent cell cycle arrest and is triggered by developmental or environmental factors and oncogene or therapy-induced stress signals^15,16^. Senescence is described solely as a tumor-suppressive mechanism, indicating that the bypass of cellular senescence is an important step in tumorigenesis^17^. Of particular interest in the context of the present study, cellular senescence is now recognized as a hallmark of GC that impacts patient outcomes and therapeutic efficacy^18^.

NUCB2 expression has a significant impact in GC^19,20^, but little is known about the exact mechanism(s) by which NUCB2 contributes to tumor progression. In the present study, we investigated NUCB2 mRNA and protein expression and the functional role of NUCB2 during progression of GC. Our data uncover a novel function of NUCB2 as an important regulator of cellular senescence in GC cells. This new activity of NUCB2 plays a significant role in aggressive tumor progression via the modulation of several other GC cellular phenotypes.

## Materials and methods

### Clinical cases

We retrospectively evaluated pathological specimens from 150 non-invasive and invasive GC patients, excluding the recurrent tumors, who had undergone surgery between 2016 and 2021, at Kitasato University Hospital, according to the criteria of the Japanese Classification of Gastric Carcinoma and the TNM classification^21,22^. Of 150 GC cases including 20 pT1, 31 pT2, 43 pT3, and 56 pT4, none of the cases had chemotherapy before gastrectomy, whereas 72 cases of 130 invasive (pT2/pT3/pT4) GC had received post-operative chemotherapy with administration of a combination of docetaxel, TS-1, oxaliplatin, capecitabine, or nibolumab. Ten samples of normal gastric tissues adjacent to GC were also investigated. All tissues were routinely fixed in 10% formalin and processed for embedding in paraffin wax, as described previously^23,24^. This study was approved by the Kitasato University Medical Ethics Committee (B21-184).

### Antibodies and other reagents

Anti-vimentin, anti-Snail, and anti-Slug antibodies were purchased from Cell Signaling Technology (Danvers, MA, USA). Anti-NUCB2, anti-Twist1, and anti-Ki-67 antibodies were obtained from Abcam (Cambridge, MA, USA). Anti-E-cadherin, anti-p21^waf1^, anti-BCL2, and anti-cyclin D1 antibodies were from Dako (Copenhagen, Denmark). Anti-p27^kip1^, anti-Rb, anti-X-linked inhibitor of apoptosis (XIAP), anti-BAX, and anti-N-cadherin antibodies were from BD Biosciences (San Jose, CA, USA). Anti-ZEB1 and anti-β-actin antibodies and adriamycin (ADR: D1515) were from Sigma-Aldrich Chemicals (St. Louis, MO, USA). Anti-phospho (p) Rb at Ser807/811, anti-cleaved caspase-3, and anti-poly (ADP-ribose) polymerase 1 (PARP1) antibodies were purchased from Cell Signaling Technology (Danvers, MA, USA). Anti-cyclin A2 and anti-cyclin B1 antibodies were from Novocastra (Newcastle, UK) and Santa Cruz Biotech (Santa Cruz, CA, USA), respectively.

### Immunohistochemistry (IHC) staining and analysis

IHC was performed using a combination of the microwave oven heating and polymer immunocomplex (Envision, Dako) methods as described previously^23,24^. Rabbit sera instead of primary antibodies were used as negative controls.

For evaluation of IHC findings, the cytoplasmic NUCB2 immunoreactivity score was derived by multiplying the percentage of immunopositive cells by the average cytoplasmic immunointensity, as described previously^23,24^. Briefly, the percentage of immunopositive cells in the total tumor cell population was subdivided into five categories as follows: 0, all negative; 1, < 30% positive cells; 2, 30–50%; 3, 50–70%; and 4, > 70%. The immunointensity was also subclassified into four groups, as follows: 0, negative; 1+, weak; 2+, moderate; and 3+, strong (Supplementary Fig. S1A). IHC scores were produced by multiplication of the two values. NUCB2 immunoreactivity was also subdivided into two categories, high and low IHC score groups, based on whether they fell above or below the cut-off value based on mean score (9.19), which was calculated by dot plot analysis (Supplementary Fig. S1B). In addition, nuclear Ki-67 immunopositivity was counted for at least 500 tumor cells and the labeling indices (LIs) were then calculated as a percentage, as described previously^25^.

### Detection of apoptotic cells

Apoptotic cells were identified in hematoxylin–eosin (HE)-stained sections according to the criteria of Kerr et al.^26^. Areas of severe inflammatory cell infiltration and necrosis were excluded because the status of some cells was ambiguous in such lesions. The number of apoptotic cells was calculated in three randomly selected high-power fields as described previously^27^.

### Cell lines and plasmids

Four GC cell lines, KE-39 (RCB1434), MKN7 (RCB3687), MKN45 (RCB1001), and MKN74 (RCB1002), were obtained from the RIKEN BioResource Research Center (Ibaraki, Japan). The cells were used within 6 months of thawing and were periodically authenticated by monitoring of cell morphology and growth curve analysis.

The NUCB2-KO cell line was generated using MKN74 cells (which have high NUCB2 expression: Supplementary Fig. S1A), as described previously^24^. Briefly, the guide RNA sequence (gRNA: 5′-GAGAAGGGTCCGAACGGCTACGG-3′) was used. The complementary oligonucleotides for gRNA were annealed and cloned into pSpCas9n(BB)-2A-Puro (PX459) V2.0 (#62988) (Addgene, Watertown, MA, USA). The pSpCas9n(BB)-2A-Puro (PX459) V2.0/gRNA construct was transfected into MKN74 cells using LipofectAMINE PLUS (Invitrogen, Carlsbad, CA, USA) to establish the NUCB2-KO lines.

### Western blot assays

Total cellular proteins were isolated using RIPA buffer [20 mM Tris–HCl (pH 7.2), 1% Nonidet P-40, 0.5% sodium deoxycholate, 0.1% sodium dodecyl sulfate]. Aliquots of the proteins were resolved by SDS-PAGE, transferred to PVDF membranes, and probed with primary antibodies coupled to the ECL detection system (GE Healthcare, Buckingham-shire, UK), as described previously^23,24^. To examine the ratios of BCL2 relative to BAX, the signals were analyzed using ImageJ software version 1.41 (NIH, Bethesda, MD; http://imageJ.nih.gov/ij). The reconstructed images of all blots with membrane edges visible are accessible in the Supplementary Materials, because some of the original full-length blots were cut prior to hybridization with antibodies.

### Flow cytometry

Cells were fixed using 70% alcohol and stained with propidium iodide (Sigma) for cell cycle analysis. The cells were then analyzed using flow cytometry on a BD FACS Calibur (BD Biosciences) with CellQuest Pro software version 3.3 (BD Biosciences), as described previously^23,24^.

### Wound healing assay

Cells were seeded into 24-well tissue culture plates, and grown to reach almost total confluence. After a cell monolayer formed, a wound was scratched with a sterile 200-μl tip. The area of the wound was also analyzed using ImageJ software version 1.41 (NIH). Cell migration parameters were calculated in pixels as wound closure, as described previously^23,24^.

### Migration assay

Cell migration was determined using 24-well transwell chambers with an 8-μm pore size (Corning, NY, USA). The lower chamber was filled with medium containing 10% serum. Cells were suspended in serum-free upper medium and added into the upper chamber. After 24 h, cells on the bottom -surface of the polycarbonate membranes were stained HE and counted using a light microscope as described previously^23,24^.

### RNAscope assay for NUCB2 mRNA in situ hybridization (ISH)

Expression of NUCB2 mRNA was analyzed in 21 GC cases using an RNAscope assay (Advanced Cell Diagnostics, Hayward, CA, USA) according to the manufacturer’s instructions. The hybridization was performed with targeted probes: Hs-NUCB2-C1(#1157401-C1), a positive control probe (#2010684), and a negative control probe (#310043) for 2 h at 40 °C. Numbers of intracytoplasmic in situ hybridization signals were counted in at least 50 cells and were then expressed as an average number of signals per cell as described previously^23^.

NUCB2 mRNA signals were divided into high and low ISH signal score groups, based on whether their average ISH signal values (fell above or below the mean of 4.3).

### Senescence-associated β-galactosidase (SA-β-gal) assay

Cells were stained for SA-β-gal activity as described previously^28^. At least 200 cells were evaluated for SA-β-gal staining and the labeling indices (LIs) were then calculated as a percentage as described previously^28^.

### Statistical analysis

Comparative data were analyzed using the Mann–Whitney *U*-test, Chi-square test, and Spearman’s correlation coefficient. Overall survival (OS) was calculated as the time between onset and death or the date of the last follow-up evaluation. Progression-free survival (PFS) was also examined from the onset of treatment until relapse, disease progression, or last follow-up evaluation. OS and PFS were estimated using the Kaplan–Meier method, and statistical comparisons were made using the logrank test. Univariate and multivariate analyses were also performed using the Cox proportional hazards regression model. The cut-off for statistical significance was set as *P* < 0.05, as described previously^23,24^.

### Ethics approval statement

This study was conducted in accordance with the principles expressed in the Declaration of Helsinki 1964 and later versions. This study was approved by the Kitasato University Medical Ethics Committee (B21-184). This article does not contain any studies with animals performed by any of the authors.

### Informed consent statement

Informed consent was obtained from all subjects involved in the study.

## Results

### NUCB2 expression in normal tissue and tumoral lesions of the stomach

**Figure 1:**
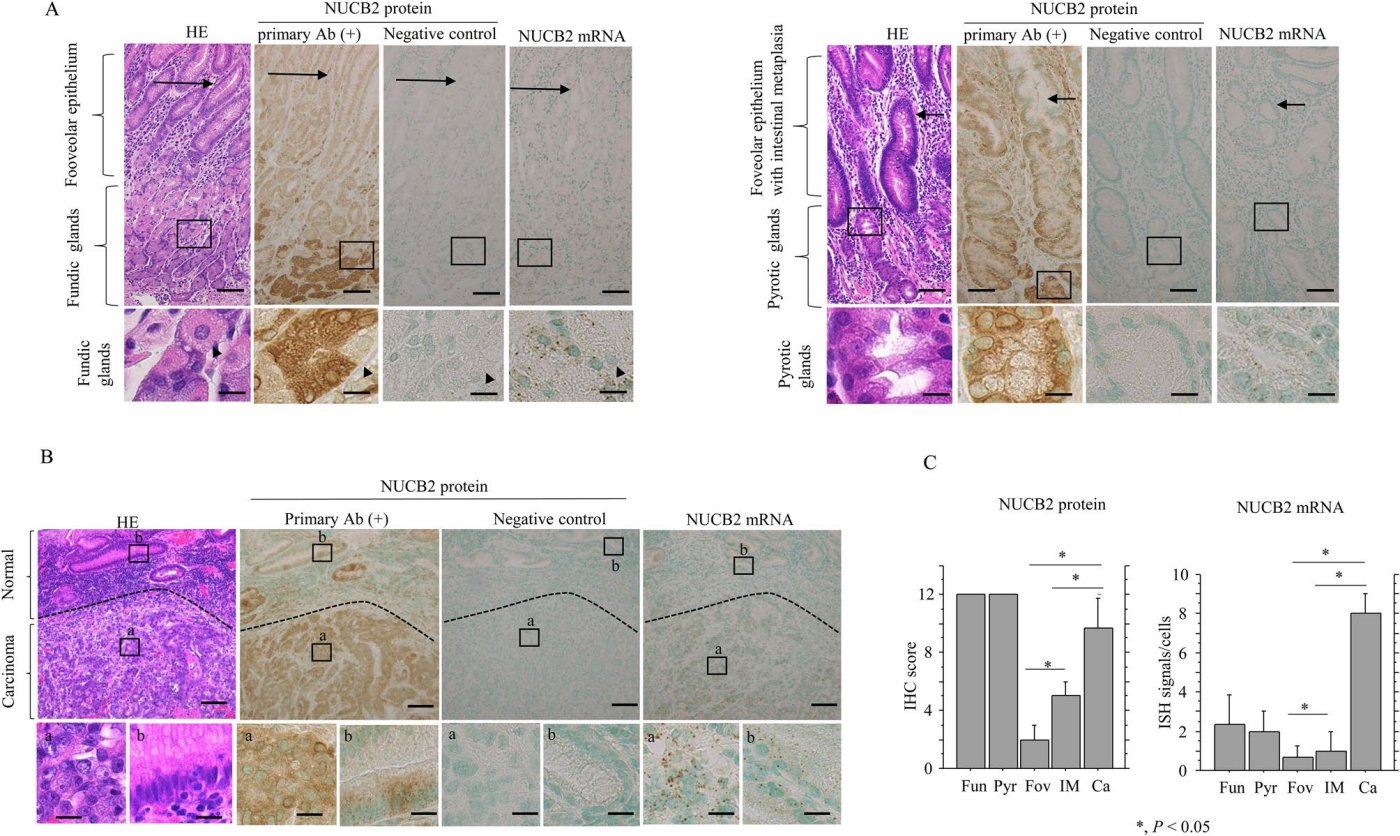
NUCB2 expression in normal and malignant gastric tissues. (**A**) Staining with HE (left) and IHC (middle) and ISH (right) for NUCB2 in fundic (left) and pyloric glandular components (right). Note the strong NUCB2 immunoreactivity in chief but not parietal cells (indicated by arrowheads) and mucous cells of fundic and pyloric glands, respectively, in contrast to the absent or low mRNA signals. Weak and moderate NUCB2 immunoreactions are also observed in foveolar epithelial cells (indicated by the long arrow to the left) in fundic glandular lesions and intestinal metaplastic epithelial cells (indicated by the short arrow to the right) in pyloric glandular areas. The closed boxes in the upper panels are magnified in the lower panels. Original magnification, × 100 (upper) and × 400 (lower). Scale bar = 50 μm (upper panels) and 20 μm (lower panels). (**B**) Staining with HE (left) and IHC (middle) and ISH (right) for NUCB2 in the carcinoma tissue adjacent to a normal tissues. Note the strong immunoreactivity and mRNA signals for NUCB2 in the carcinoma as compared to the normal tissues. Closed boxes in the upper panels are magnified in the lower panels. Original magnification, × 40 (upper) and × 400 (lower). Scale bar = 100 μm (upper) and 20 μm (lower). (**C**) IHC scores (left) and ISH signal scores (right) for NUCB2 in fundic (Fu) and pyloric glands (Py), foveolar (Fov), intestinal metaplasia (IM), and carcinoma (Ca) tissues. The values shown are means ± SDs. Statistical analyses were carried out using the Mann–Whitney *U*-test.

In normal gastric lesions, strong cytoplasmic NUCB2 immunoreactivity was found in chief (but not parietal) and mucinous cells of the fundic and pyloric glands, respectively, whereas lower or moderate NUCB2 immunoreactivities were also observed in foveolar and intestinal metaplastic epithelial cells; however, intracytoplasmic dots of NUCB2 mRNA were either rare or absent in the lesions (Fig. 1A). In contrast, strong cytoplasmic NUCB2 immunoreactivity and abundant mRNA signals were observed in GC tissues as compared to normal tissues (Fig. 1B). There was a significant stepwise increase in NUCB2 IHC and ISH signal scores from foveolar through intestinal metaplastic epithelial to GC lesions. In contrast, there was no correlation between these signals in fundic and pyloric glandular components (Fig. 1C). These findings suggest that NUCB2 expression is transcriptionally upregulated in GC, whereas it is also post-translationally stabilized in fundic and pyloric glandular components.

### NUCB2 is an independent prognostic factor in GC

**Figure 2:**
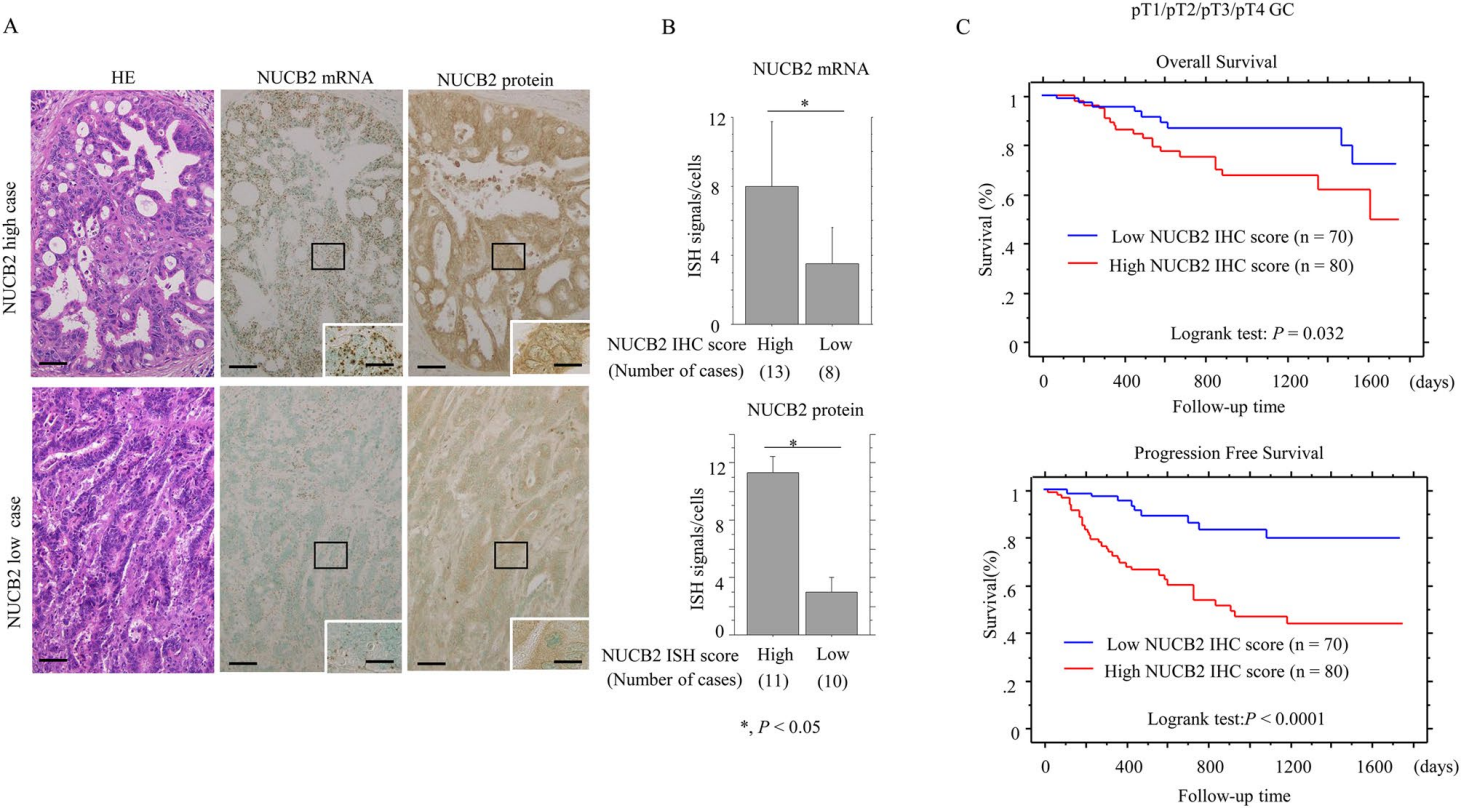
Upregulation of NUCB2 is associated with poor prognosis in GC. (**A**) Staining with HE (left) and ISH (middle) and IHC (right) for NUCB2 in cases with high (upper) and low (lower) NUCB2 expression. Note the association between NUCB2 mRNA and protein expression in GC. Closed boxes are magnified in the insets. Original magnification, × 100 and × 400 (insets). Scale bar = 50 μm and 20 μm (insets). (**B**) Upper: NUCB2 mRNA signal scores between the high and low IHC score categories. Lower: NUCB2 IHC scores between the high and low ISH signal score categories. The values shown are means ± SDs. Statistical analyses were carried out using the Mann–Whitney *U*-test. (**C**) The associations between NUCB2 IHC scores and prognosis in GC. OS (upper) and PFS (lower) relative to NUCB2 IHC scores using mean scores as cut-off values. n, number of cases. Statistical analyses were performed using the logrank test.

**Figure 3:**
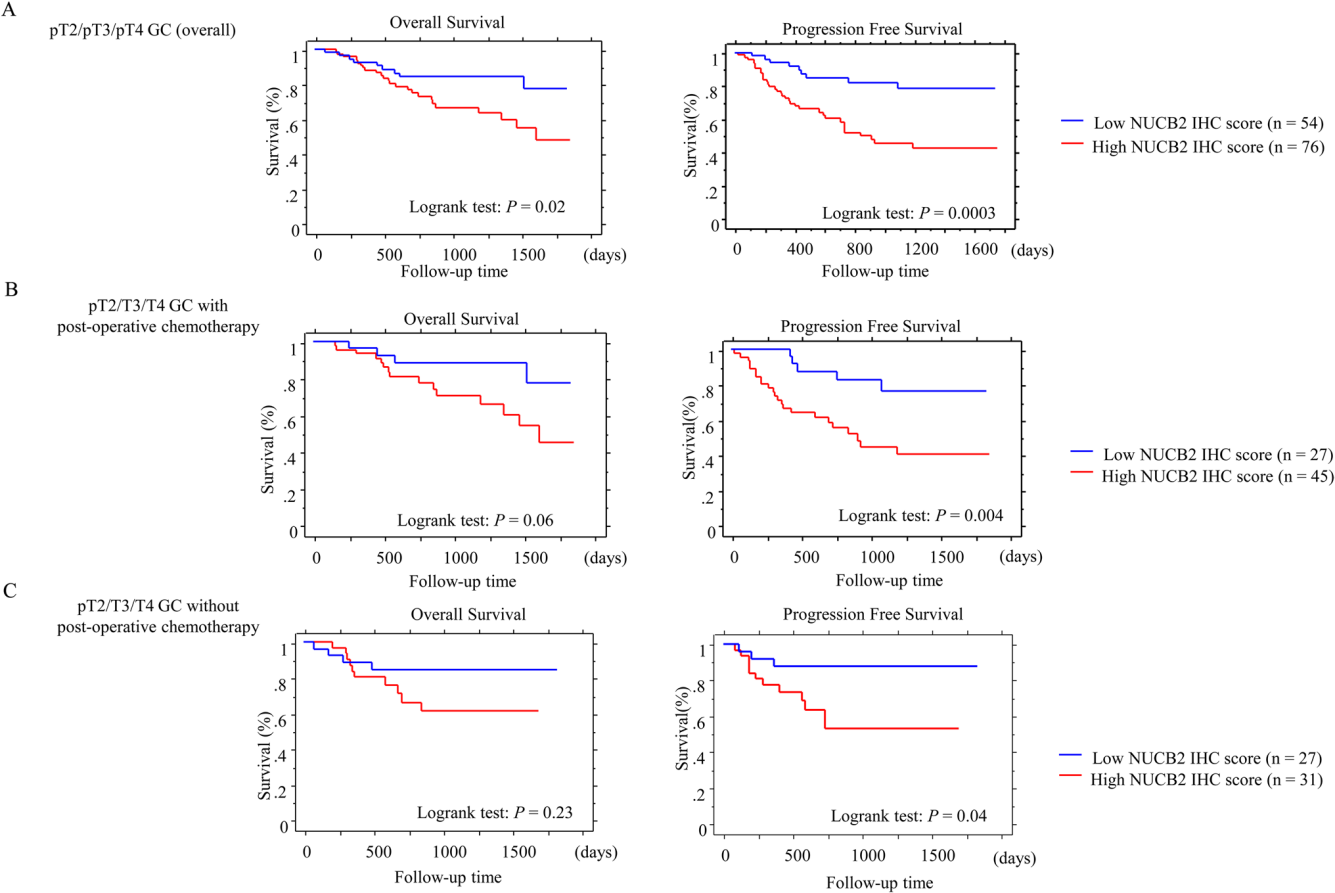
Upregulation of NUCB2 is associated with poor prognosis in invasive GC. The associations between NUCB2 IHC scores and prognosis in all of invasive GC (**A**), invasive GC with and without post-operative chemotherapy (**B** and **C**, respectively). OS (left) and PFS (right) relative to NUCB2 IHC scores using mean scores as cut-off values. n, number of cases. Statistical analyses were performed using the logrank test.

Representative ISH and IHC images of NUCB2 in GC are shown in Fig. 2A. NUCB2 mRNA signal scores were significantly higher in the NUCB2 IHC-high compared to the NUCB2 IHC-low score groups; this was consistent with the highest NUCB IHC scores being in the NUCB ISH-high score group (Fig. 2B). In 150 non-invasive (pT1) and invasive (pT2/pT3/pT4) GC, high NUCB2 IHC scores were significantly associated with unfavorable prognostic factors including deep invasion, lymph-vascular tumor invasion, lymph node metastasis, and advanced clinical stage (Table 1). Kaplan–Meier analysis showed that the NUCB2 IHC-high score group had shorter OS and PFS than the low score group (Fig. 2C). Universal Cox progression hazards regression revealed that NUCB2 IHC scores, as well as several clinicopathological factors, had a significant impact on OS and PFS. Multivariate Cox regression analysis also showed that NUCB2 IHC score and tumor venous invasion were independent prognostic factors for PFS but not OS (Table 2). In 130 invasive (pT2/pT3/pT4) GC, NUCB IHC-high score was also an independent prognostic factor for PFS that correlated with aggressive tumor behavior (Fig. 3A and Supplementary Table S2). Similar findings were also observed in 72 invasive GC cases in which patients received post-operative chemotherapy (Fig. 3B and Table 3), but not in the 58 invasive tumors from patients who did not receive chemotherapy (Fig. 3C and Supplementary Table S3).

**Table 1:** Relationship between NUCB2 expression and clinicopathologic factors in non-invasive and invasive gastric carcinoma.

Clinicopathologic factors	n	NUCB2 immunoreactivity: n (%)		*P*-value
		High score ≥ 10	Low score < 10	
Gender				
Female	42	18 (42.9)	24 (57.1)	0.1
Male	108	62 (57.4)	46 (42.6)	
Age				
≥ 70 years	88	50 (56.8)	38 (43.2)	0.3
< 70 years	62	30 (48.4)	32 (51.6)	
Location				
L	58	30 (51.7)	28 (48.3)	0.8
U, M	92	50 (54.3)	42 (45.7)	
Tumor size				
≥ 68 mm	57	34 (59.6)	23 (40.4)	0.2
< 68 mm	93	46 (49.5)	47 (50.5)	
Histological type				
Well/moderate	77	41 (53.2)	36 (46.8)	0.9
Poor	73	39 (53.4)	34 (46.6)	
pT factor				
pT1, pT2	51	20 (39.2)	31 (60.8)	< 0.05
pT3, pT4	99	60 (60.6)	39 (39.4)	
Lymphatic invasion				
Positive	84	54 (64.3)	30 (35.7)	< 0.01
Negative	66	26 (39.4)	40 (60.6)	
Venous invasion				
Positive	119	73 (61.3)	46 (38.7)	< 0.01
Negative	31	7 (22.6)	24 (77.4)	
LN metastasis				
Positive	78	51 (65.4)	27 (34.6)	< 0.01
Negative	72	29 (40.3)	43 (59.7)	
Pathological stage				
I, II	90	40 (44.4)	50 (55.6)	< 0.05
III, IV	60	40 (66.7)	20 (33.3)	

n, number of cases; U, upper; M, middle; L, lower; LN, lymph node; well/mod, well/moderately differentiated; poor, poorly differentiated.

Cut-off value is defined as mean value (9.19) of NUCB2 score.

Statistical analyses were carried out using the Chi-square test.

pT factor and pathological stage refer to the criteria of the Japanese Classification of Gastric carcinoma and TNM classification.

**Table 2:** Univariate and multivariate analysis for overall survival and progression-free survival in non-invasive and invasive gastric carcinoma.

Univariate analysis					Multivariate analysis				
Variables	Cut-off	Log rank c2	*P*-value	Unfavorable factor	Variable	Cut-off	Hazard Ratio	95% CI	*P*-value
Overall survival					Overall survival				
Gender	Male/female	1.394	0.3		Age (years)	69/70	2.168	0.94–4.99	0.06
Age (years)	69/70	2.462	0.02	> 70	Lymphatic invasion	−/+	0.48	0.48–4.04	0.5
Location	U, M/L	1.783	0.1		Venous invasion	−/+	0.489	0.48–31.03	0.1
Tumor size (mm)	67/68	1.874	0.08		LN metastasis	−/+	0.241	0.24–3.87	0.9
Histology type	Well, moderate/poor	0.601	0.1		Pathological stage	I,II/III,IV	0.089	0.08–1.06	0.06
Depth	pT1, pT2/pT3, pT4	0.348	0.05		NUCB2 expression	Low/High	0.706	0.70–3.59	0.2
Lymphatic invasion	−/+	3.266	0.009	+					
Venous invasion	−/+	8.642	0.03	+					
LN metastasis	−/+	3.445	0.006	+					
Post-operative chemotherapy	None/done	0.095	0.7						
Pathological stage	I, II/III, IV	0.248	0.0008	III, IV					
NUCB2 expression	Low/High	2.301	0.03	High expression					
Progression-free survival					Progression-free survival				
Gender	Male/female	1.281	0.4		Tumor size (mm)	67/68	1.865	0.94–3.68	0.07
Age (years)	69/70	1.73	0.08		Depth	pT1, pT2/pT3, pT4	0.809	0.26–2.48	0.7
Location	U, M/L	1.234	0.4		Lymphatic invasion	−/ +	1.449	0.58–3.57	0.4
Tumor size (mm)	67/68	3.012	0.0004	> 68	Venous invasion	−/ +	8.009	1.05–60.75	0.04
Histology type	Well, moderate/poor	0.856	0.6		LN metastasis	−/ +	0.697	0.2–2.42	0.5
Depth	pT1, pT2/pT3, pT4	0.252	0.003	pT3, pT4	Pathological stage	I, II/III, IV	0.352	0.1–1.12	0.07
Lymphatic invasion	−/+	3.86	0.0006	+	NUCB2 expression	Low/High	2.649	1.25–5.6	0.01
Venous invasion	−/+	13.391	0.01	+					
LN metastasis	−/+	3.804	0.0004	+					
Post-operative chemotherapy	None/done	0.931	0.3						
Pathological stage	I, II/III, IV	0.219	< 0.0001	III, IV					
NUCB2 expression	Low/High	4.092	0.0002	High expression					

LN, lymph node; U, upper; M, middle; L, lower; well, mod, well and moderately differentiated; poor, poorly differentiated; Cut-off value is defined as the mean value (9.19) of NUCB2 score.

pT factor and pathological stage refer to the criteria of the Japanese Classification of Gastric Carcinoma and TNM classification.

**Table 3:** Univariate and multivariate analysis for overall survival and progression-free survival in invasive gastric carcinoma receiving post-operative chemotherapy.

Univariate analysis					Multivariate analysis				
Variables	Cut-off	Log rank c2	*P*-value	Unfavorable factor	Variable	Cut-off	Hazard Ratio	95% CI	*P*-value
Overall survival					Overall survival				
Gender	Male/female	0.594	0.4		Histology type	Well, moderate/poor	0.407	0.11–1.44	0.1
Age (years)	69/70	0.042	0.8		Lymphatic invasion	−/+	2.724	0.28–26.21	0.3
Location	U, M/L	0.002	0.9		Pathological stage	I, II/III, IV	0.424	0.07–2.26	0.3
Tumor size (mm)	67/68	1.98	0.1						
Depth	pT2/pT3,pT4	1.484	0.2						
Histology type	Well, moderate/poor	4.824	0.02	Poor					
Lymphatic invasion	−/+	4.189	0.04	+					
Venous invasion	−/+	–	–						
LN metastasis	−/+	–	–						
Pathological stage	I, II/III, IV	5.682	0.01	III, IV					
NUCB2 expression	Low/High	3.443	0.06						
Progression-free survival					Progression-free survival				
Gender	Male/female	0.126	0.72		Tumor size (mm)	67/68	1.806	0.74–4.39	0.1
Age (years)	69/70	0.002	0.9		Pathological stage	I, II/III, IV	0.36	0.11–1.13	0.08
Location	U, M/L	0.041	0.8		NUCB2 expression	Low/High	3.359	1.36–9.5	0.009
Tumor size (mm)	67/68	5.591	0.01	> 68					
Depth	pT2/pT3,pT4	3.389	0.06						
Histology type	Well, moderate/poor	0.084	0.7						
Lymphatic invasion	−/+	2.543	0.1						
Venous invasion	−/+	ND	ND						
LN metastasis	−/+	1.644	0.1						
Pathological stage	I, II/III, IV	7.093	0.007	III, IV					
NUCB2 expression	Low/High	8.105	0.004	High expression					

LN, lymph node; U, upper; M, middle; L, lower; well, mod, well and moderately differentiated; poor, poorly differentiated; cut-off value is defined as the mean value (9.19) of NUCB2 score.

pT factor and pathological stage refer to the criteria of the Japanese Classification of Gastric Carcinoma and TNM classification.

These findings suggest that high NUCB2 is independent prognostic factor that is correlated with aggressive GC behavior, particularly in patients with invasive GC who have received post-operative chemotherapy.

### Knockout of NUCB2 decreases proliferation, apoptosis, and migration through induction of cellular senescence in GC cells

High levels of endogenous NUCB2 expression were found in all of four GC cell lines studied (Supplementary Fig. S2A). We established NUCB2-KO cell line clones (MK74-NUCB2-KO#91 and #92) using MKN74 cells, based on the findings that MKN74 proliferated more rapidly and more closely resembled a GC cell in terms of morphology (Supplementary Fig. S2B).

**Figure 4:**
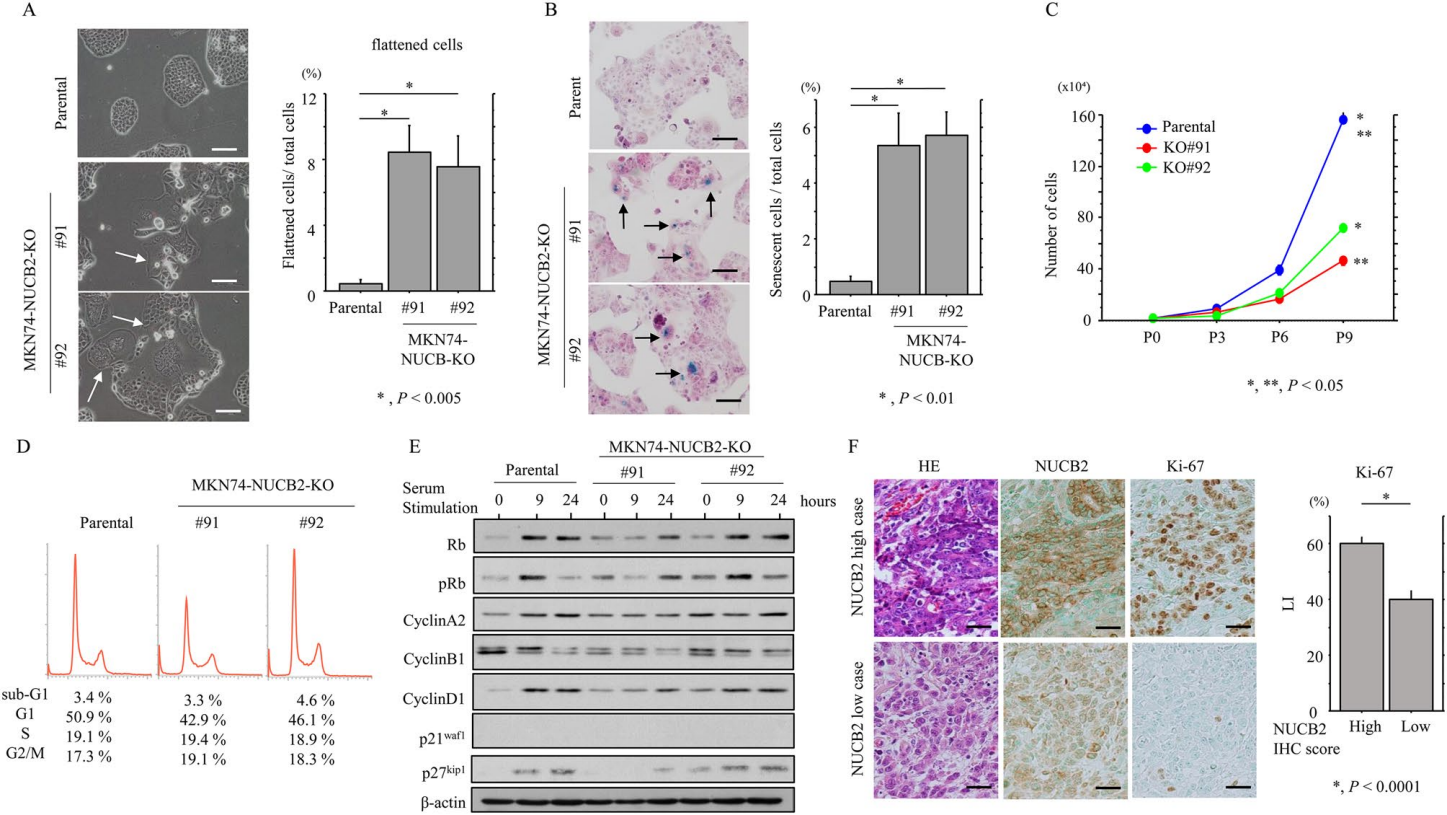
Changes in morphology and proliferation following NUCB2 knockout in GC cells. (**A**) Left: phase contrast images of NUCB2-KO cells, revealing the switch towards a flattened morphology. Scale bar = 30 μm. Right: number of flattened cells is calculated as a percentage of all cells. The values shown are means ± SDs. Statistical analyses were carried out using the Mann–Whitney *U*-test. (**B**) Left: SA-β-gal assay for NUCB2-KO and parental cells. Note the blue aggregates in the cytoplasm of flattened cells (senescent cells: indicate by arrows). Original magnification, × 400. Scale bar = 30 μm. Right: labeling indices for SA-β-gal positive cells are calculated as a percentage. The values shown are means ± SDs. Statistical analyses were carried out using the Mann–Whitney *U*-test. (**C**) NUCB2-KO and parental cells were seeded at low density. Cell numbers are presented as means ± SDs. P0, P3, P6, and P9 are 0, 3, 6, and 9 days after seeding, respectively. The experiments were performed in triplicate. Statistical analyses were carried out using the Mann–Whitney *U*-test. (**D**) Flow cytometry analysis of NUCB2-KO and parental cells 3 days after seeding (P3). The experiments were performed in triplicate. (**E**) Western blot analysis for the indicated proteins in total lysates from NUCB2-KO and parental cells following re-stimulation of serum-starved (24 h) cells with 10% serum for the indicated times. (**F**) Left: staining with HE and IHC for the indicated proteins in GC. Original magnification, × 200. Scale bar = 30 μm. Right: labeling indices for Ki-67 positive cells are demonstrated as a percentage. The values shown are means ± SDs. Statistical analyses were carried out using the Mann–Whitney *U*-test.

The MKN74-NUCB2-KO cells demonstrated a significant switch towards a flattened, senescence-like morphology (Fig. 4A), consistent with a significant increase in the number of SA-β-gal-positive cells (Fig. 4B). The NUCB2-KO cells also tended to proliferate more slowly, particularly in the exponential growth phase (Fig. 4C), and appeared to have more or less cells in G1 and G2/M phages of cell cycle than parental cells, respectively, although the difference was not statistically significant due to the small number (n = 3) of experiments (Fig. 4D).

We also performed serum starvation-release experiments to examine the expression of cell cycle-related molecules. At 9 h or 24 h after release into the cell cycle (as compared to 0 h), the expression of Rb, cyclin A2, cyclin B1, cyclin D1 and p27^kip1^ was substantially decreased in NUCB2-KO#91 cells relative to parental cells, whereas expression of p21^waf1^, which is often upregulated during senescence^29^, was absent (Fig. 4E and Supplementary Fig. S3). In line with these findings, Ki-67 LIs were significantly higher in the GC NUCB2 IHC-high score group as compared to the NUCB2 IHC-low score group (Fig. 4F).

**Figure 5:**
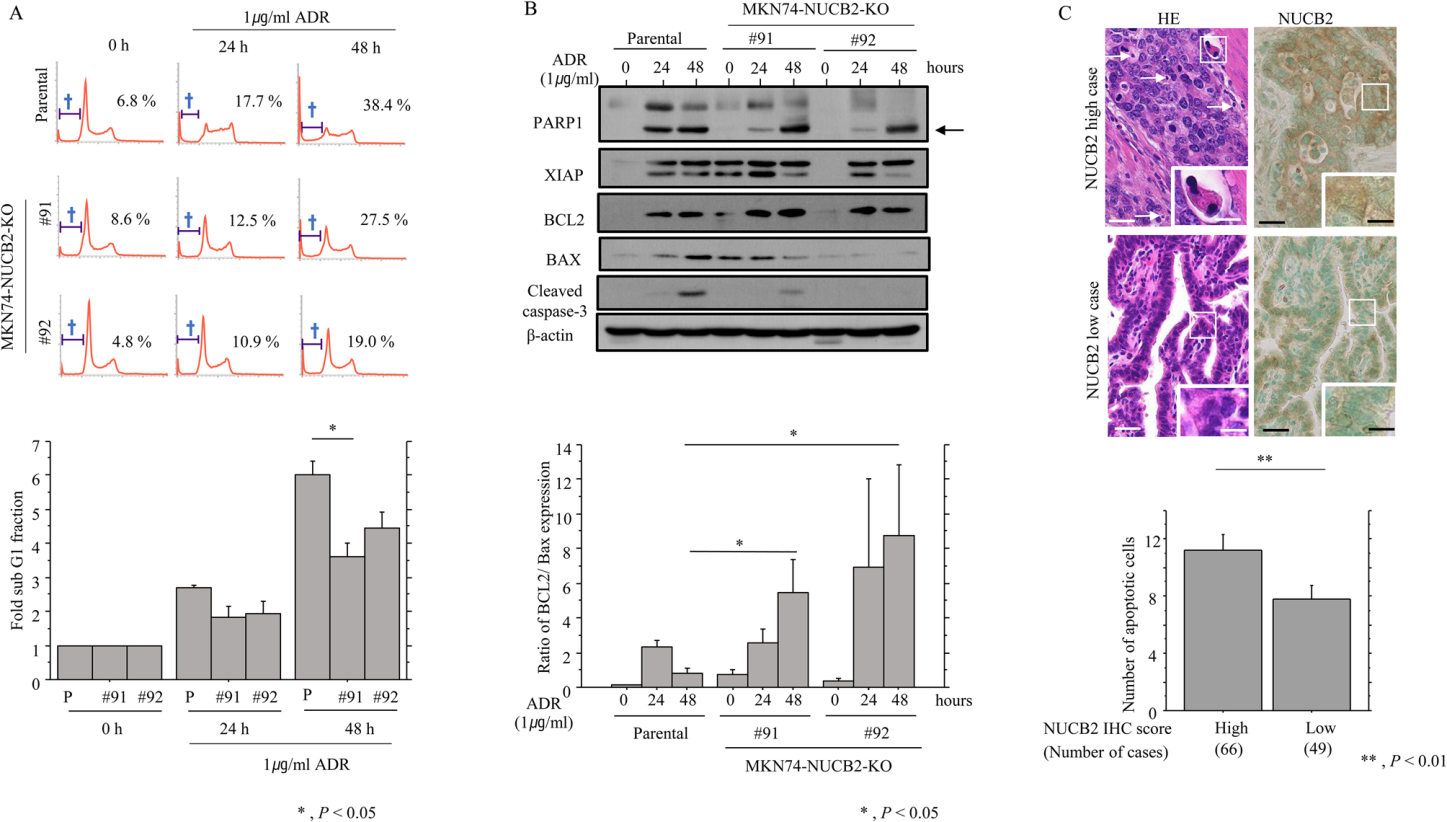
Changes in susceptibility to apoptosis following NUCB2 knockout in GC cells (**A**) upper: NUCB2-KO and parental cells were treated with 1 μg/mL Adriamycin (ADR) for the times shown. Daggers indicate sub-G1 fractions. The experiment was performed in triplicate. Lower: the percentage of cells undergoing apoptosis (sub-G1) was calculated following flow cytometry. The values shown are means ± SDs. Statistical analyses were carried out using the Mann–Whitney *U*-test. P, Parental. (**B**) Upper: western blot analysis for the indicated proteins in NUCB2-KO and parental cells after 1 μg/mL ADR for the times shown. Cleaved PARP1 is indicated by an arrow. Lower: values of endogenous BCL2 relative to BAX protein were calculated by normalization to β-actin after 1 μg/mL ADR for the times shown. The experiment was performed in triplicate. The values shown are means ± SDs. Statistical analyses were carried out using the Mann–Whitney *U*-test. (**C**) Upper: staining with HE and IHC for NUCB2 in GC. Note the many apoptotic cells (indicated by arrows) in GC cases with strong NUCB2 immunoreactivity. The closed boxes are magnified in the insets. Original magnification, × 200 and × 400 (insets). Scale bar = 30 μm and 20 μm (insets). Lower: the number of apoptotic cells in three randomly selected areas of GC. The values shown are means ± SDs. Statistical analyses were carried out using the Mann–Whitney *U*-test.

We next examined the role of NUCB2 in susceptibility to apoptosis in GC cells. When treatment with the genotoxin, ADR, NUCB2-KO cells were significantly less sensitive to apoptosis when compared to parental cells (Fig. 5A). This was consistent with decreased expression of cleaved PARP1, cleaved caspase-3, and BAX, as well as increased ratios of BCL2 relative to BAX (Fig. 5B and Supplementary Fig. S4). In addition, there were significantly more apoptotic cells in NUCB2 IHC-high cells as compared to NUCB2 IHC-low cells (Fig. 5C).

**Figure 6:**
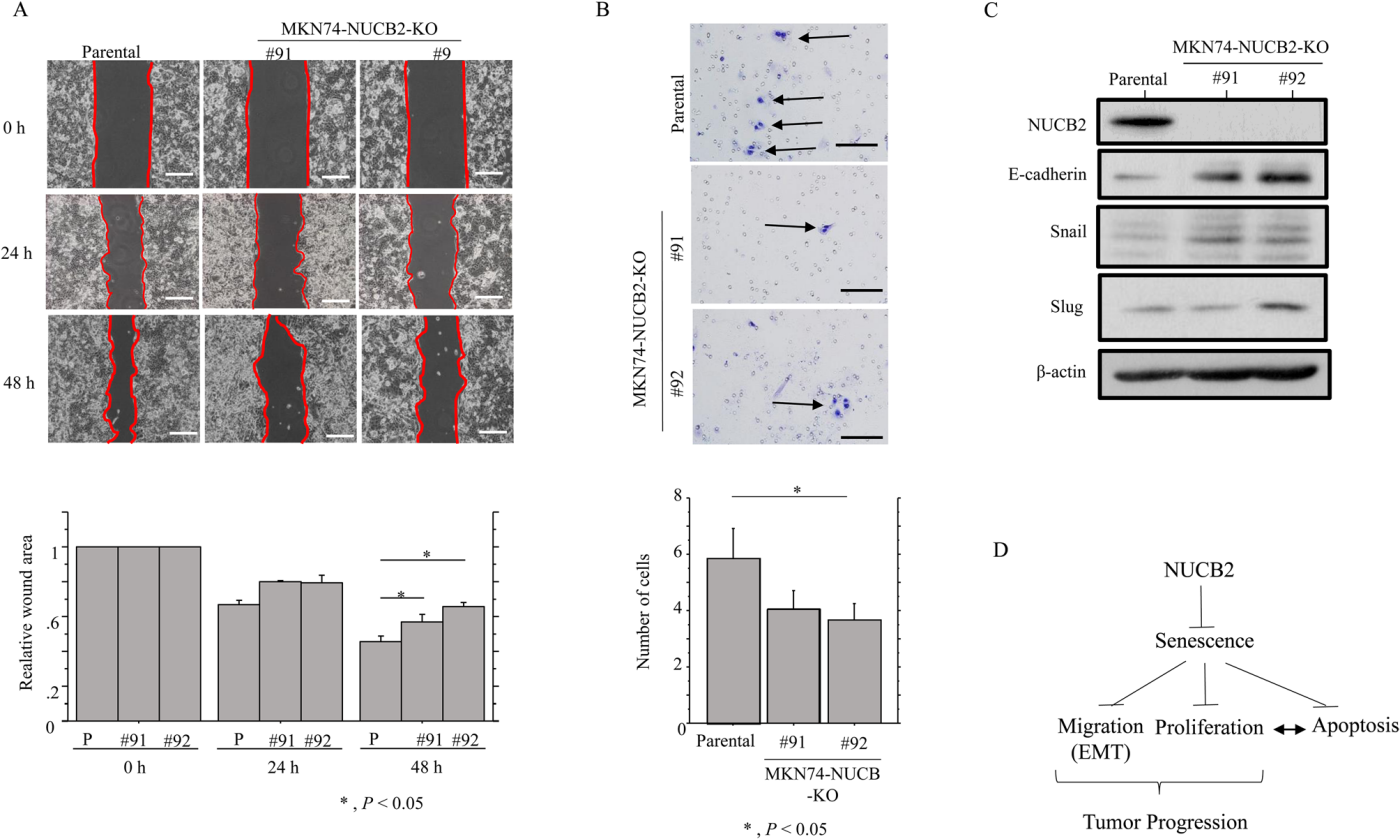
Changes in migration capability following NUCB2 knockout in GC cells. (**A**) Upper: wound-healing assay with NUCB2-KO and parental cells. Phase contrast images were taken 24 h and 48 h after the wound was made. Scale bar = 50 μm. Lower: the values of wound areas at 0 h were set as 1. The fold wound areas are presented as means ± SDs. The experiments were performed in triplicate. Statistical analyses were carried out using the Mann–Whitney *U*-test. P, Parental (**B**) Upper: NUCB2-KO and parental cells were seeded in 24-well Transwell plates and incubated for 24 h in medium without serum. Cells (indicated by arrows) were stained with HE and counted using a light microscope. Scale bar = 30 μm. Lower: numbers of migrated cells are presented as means ± SDs. The experiments were performed in triplicate. Statistical analyses were carried out using the Mann–Whitney *U*-test. (**C**) Western blot analysis for the indicated proteins in total lysates from NUCB2-KO and parental cells. The experiments were performed in duplicate. (**D**) Schematic representation of the functional role of NUCB2 during GC progression.

To further examine whether NUCB2 expression contributes to cell migration capability, we carried out scratch and migration assays. The NUCB-KO cells refilled wounded empty spaces more slowly (Fig. 6A), in line with the significantly decreased migration rates as compared to the parental cells (Fig. 6B). We also observed increased E-cadherin expression in NUCB2-KO cells, which was independent of the expression of several E-cadherin repressors including Snail, Slug, Twist1, and ZEB1 (Fig. 6C and Supplementary Fig. S5).

These findings suggest that NUCB2-KO induces cellular senescence, leading to increased E-cadherin expression and decreased proliferation, as well as reduced to susceptibility to apoptosis and attenuated migration in GC cells.

## Discussion

In the present study, we demonstrated that the high NUCB2 protein expression in chief (but not parietal) and mucinous cells of the fundic and pyloric glands, respectively, is likely due to post-translational regulation of NUCB2 expression. Given that NUCB2 is involved in a wide variety of basic cellular functions including insulin release and regulation of energy homeostasis^30^, it is possible that high NUCB2 expression may be required for normal gastric secretory function. In support of this, NUCB2 regulates gastric secretion by establishing an agonist-releasable Ca^2+^ store in the endoplasmic reticulum or Golgi apparatus^31^.

We also found that NUCB2 protein levels were significantly higher in GC as compared to foveolar and intestinal metaplastic epithelial components, and that this was most likely due to transcriptional upregulation of NUCB2 during GC onset. Moreover, a high level of cytoplasmic NUCB2 was significantly associated with several aggressive clinicopathological factors and was an independent predictor of poor PFS in patients with non-invasive and invasive GC, as well as invasive GC cases in which patients had received post-operative chemotherapy. An earlier study found an association between high nuclear NUCB2 expression and poor prognosis in GC^19^. Both studies are thus consistent with high NUCB2 being associated with poor prognosis, although there is a discrepancy regarding the subcellular localization of the protein. This may be due to the use of different antibodies for detection of NUCB2.

Importantly, knockout of NUCB2 induces senescence in GC cells, along with changes in several other cellular phenotypes. First, NUCB2 loss slows proliferation and reduces the expression of several cell cycle-related molecules. This is consistent with NUCB2 knockdown in bladder carcinoma cells, which inhibits invasion and proliferation^32^. Given that senescent cells lack proliferative capacity^15,16^, we suggest that NUCB2-dependent suppression of senescence in GC at least partially explained its role in tumorigenesis. This conclusion is supported by the finding that chemotherapy-induced senescence is associated with favorable outcomes^33^. Second, NUCB2-KO cells also prevented ADR-induced apoptosis, probably due to an increased ratio of BCL2 relative to BAX expression. This is consistent with other findings that BCL2 is one of several pro-survival factors that are upregulated in senescent cells^34^. In fact, senescent cells activate several prosurvival factors and become resistant to apoptosis^35^. Given that apoptosis might also induce a compensative proliferation under certain conditions^36^, we suggest that NUCB2 may increase susceptibility to apoptosis in GC cells through inhibition of anti-apoptotic factors, yet stimulate proliferation by blocking cellular senescence. In support of this hypothesis, we observed that both apoptotic and proliferative indices were significantly higher in the NUCB2 IHC-high score group if GC as compared to the NUCB2 IHC-low score group. However, NUCB2/nesfatin-1 induces a concentration-dependent increase in the rate of apoptosis of adrenocortical cells^13^, whereas its downregulation in renal carcinoma cell lines leads to increased apoptosis^37^. Thus, the effect of NUCB2 expression on apoptosis is tissue- and cell type-dependent.

Finally, we suggest a link between NUCB2, E-cadherin, and the epithelial to mesenchymal transition (EMT), which is a hallmark of cancer associated with reduced apoptosis and increased invasiveness^34^. NUCB2 and ZEB1 co-operate to repress E-cadherin, concomitant with increased proliferation and migration in uterine carcinosarcoma^24^. Here, we observed that NUCB2 knockout increased E-cadherin levels, and reduced migration. We therefore infer that NUCB2 overexpression may contribute to EMT and the subsequent aggressive cellular phenotypes.

## Conclusion

Our results suggest a novel role of NUCB2 in GC progression (Fig. 6D). NUCB2 expression serves as a regulator of cellular senescence and this in turn is associated with aggressive GC behaviors, leading to the modulation of proliferation, susceptibility to apoptosis, and migration.

### Supplementary Information

Supplementary Legends.

Supplementary Figure S1.

Supplementary Figure S2.

Supplementary Figure S3.

Supplementary Figure S4.

Supplementary Figure S5.

Supplementary Table S1.

Supplementary Table S2.

Supplementary Table S3.

## Data Availability

The data sets generated during and/or analyzed during the current study are available from the corresponding author on reasonable request.
