# Supplementary material for: Nucleobindin 2 inhibits senescence in gastric carcinoma
Source: Sci Rep. 2024 May 17;14:11261. doi: 10.1038/s41598-024-61111-5 (PMC11101443; doi:10.1038/s41598-024-61111-5)
Supplement: Supplementary file 1 — Supplementary Legends. [file 41598_2024_61111_MOESM1_ESM.docx]

**Legends of supplementary figures**

**Supplementary Figure S1.** (A) Staining with IHC for NUCB2 in GC, demonstrating the negative, weak, moderate, and strong NUCB2 immunointensities in GC components. Note the strong NUCB2 immunointensity in stromal cells (indicated by arrows), in contrast to negative and weak NUCB2 immunointensity in GC. Original magnification, x200. Scale bar = 30 μm. (B) Dot plot analysis for NUCB2 IHC scores in 150 GC cases.

**Supplementary Figure S2.** (A,B) Western blot analysis for the indicated proteins in total lysates from the indicated cell lines. The reconstructed images of all blots with membrane edges visible are shown because some of the original full-length bots were cut prior to hybridization with antibodies (upper) and the replicate blots (lower).

**Supplementary Figure S3.** (A,B) Original images of western blot analysis for the indicated proteins in total lysates from MKN74-NUCB2-KO and parental cells. The reconstructed images of all blots with membrane edges visible are shown because some of the original full-length bots were cut prior to hybridization with antibodies(upper) and the replicate blots (lower).

**Supplementary Figure S4.** (A,B) Original images of western blot analysis for the indicated proteins in total lysates from MKN74-NUCB2-KO and parental cells. The predictive sizes are indicated by arrows. The reconstructed images of all blots with membrane edges visible are shown because some of the original full-length bots were cut prior to hybridization with antibodies(upper) and the replicate blots (lower).

**Supplementary Figure S5.** (A,B, C) Original images of western blot analysis for the indicated proteins in total lysates from MKN74-NUCB2-KO and parental cells. The reconstructed images of all blots with membrane edges visible are shown because some of the original full-length bots were cut prior to hybridization with antibodies (upper) and the replicate blots (lower).
