# Supplementary figures and images for: Nucleobindin 2 inhibits senescence in gastric carcinoma
Source: Sci Rep. 2024 May 17;14:11261. doi: 10.1038/s41598-024-61111-5 (PMC11101443; doi:10.1038/s41598-024-61111-5)

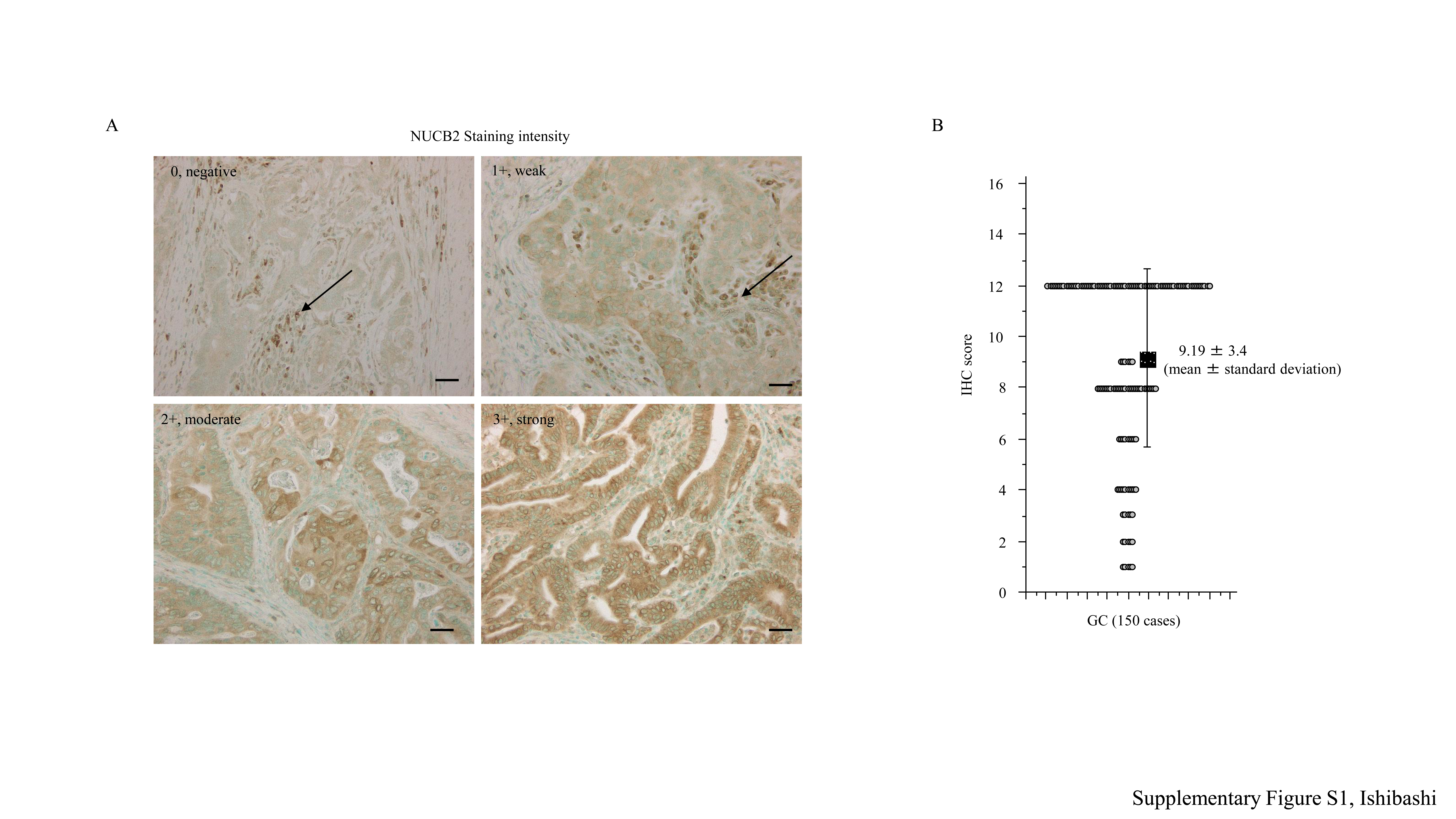

Supplement: Supplementary file 2 — Supplementary Figure S1. [file 41598_2024_61111_MOESM2_ESM.tif]

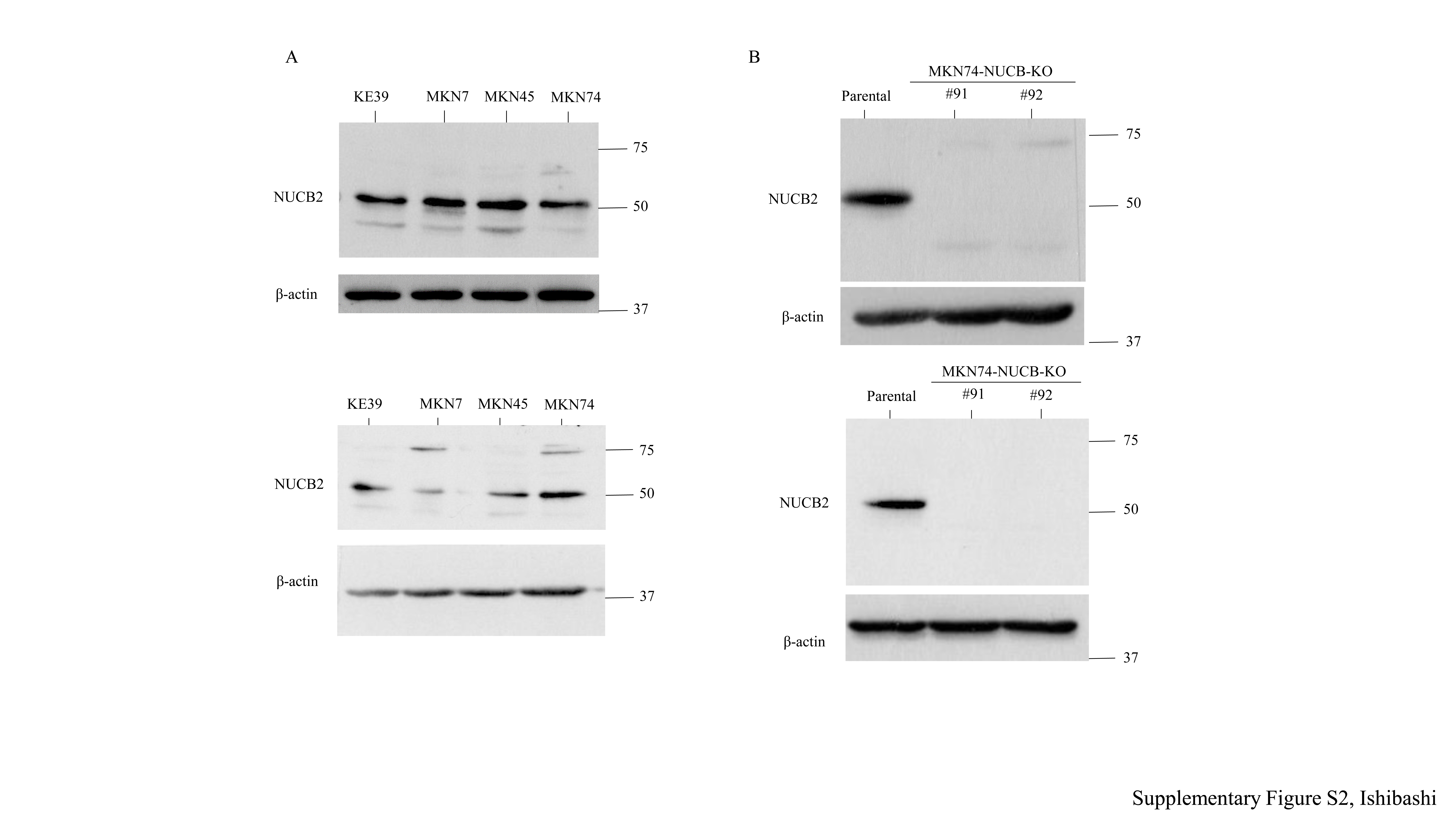

Supplement: Supplementary file 3 — Supplementary Figure S2. [file 41598_2024_61111_MOESM3_ESM.tif]

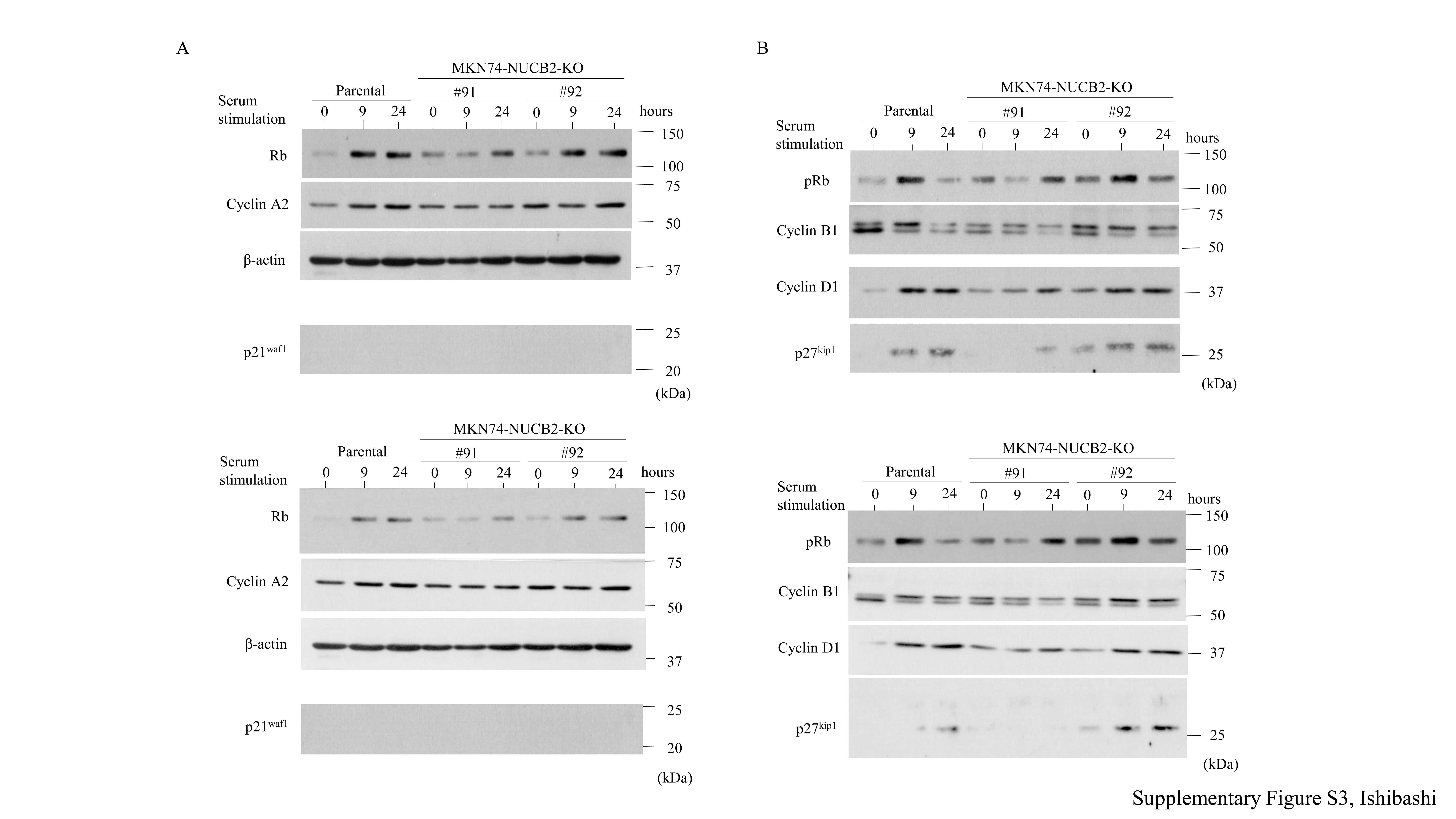

Supplement: Supplementary file 4 — Supplementary Figure S3. [file 41598_2024_61111_MOESM4_ESM.tif]

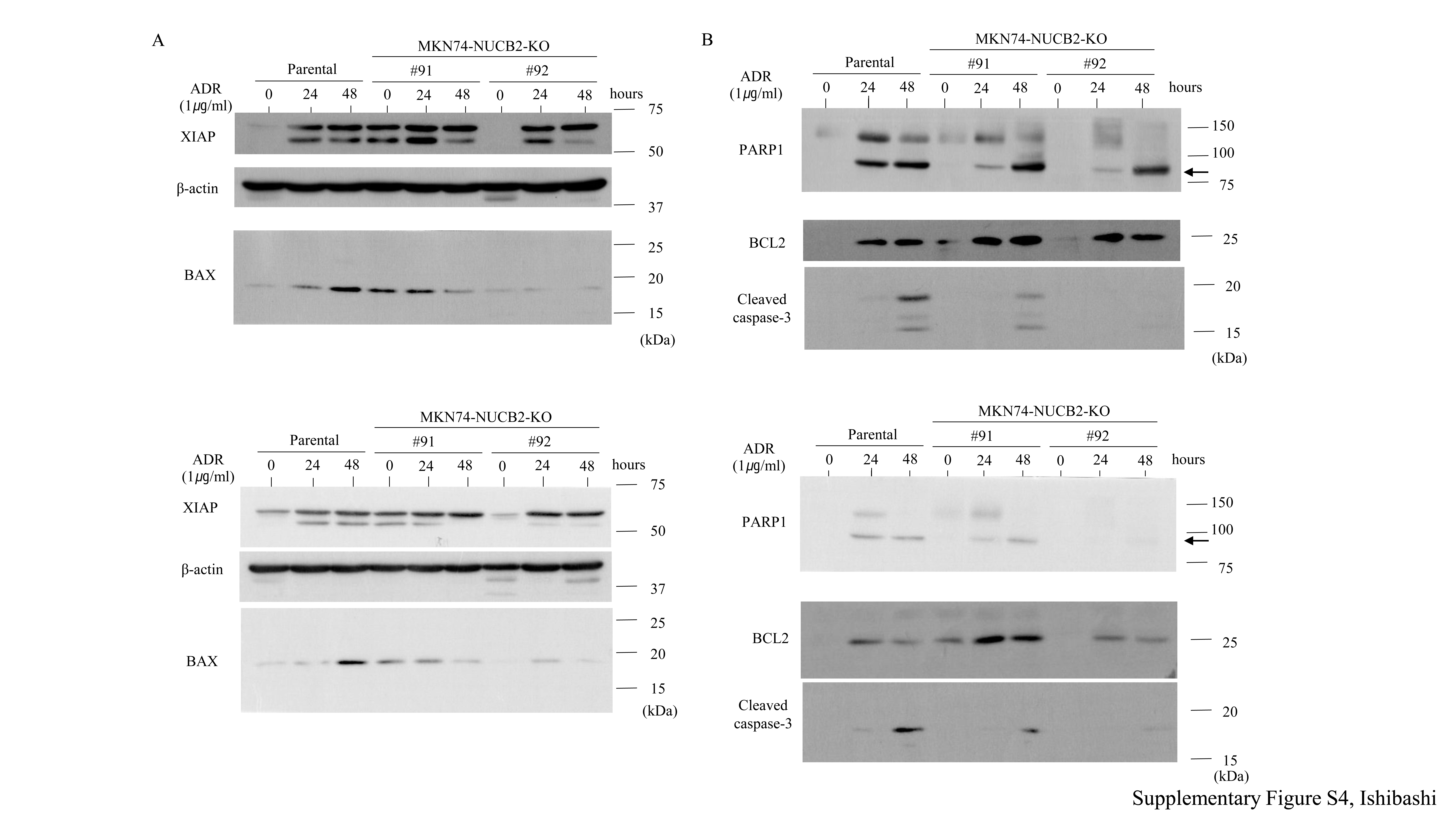

Supplement: Supplementary file 5 — Supplementary Figure S4. [file 41598_2024_61111_MOESM5_ESM.tif]

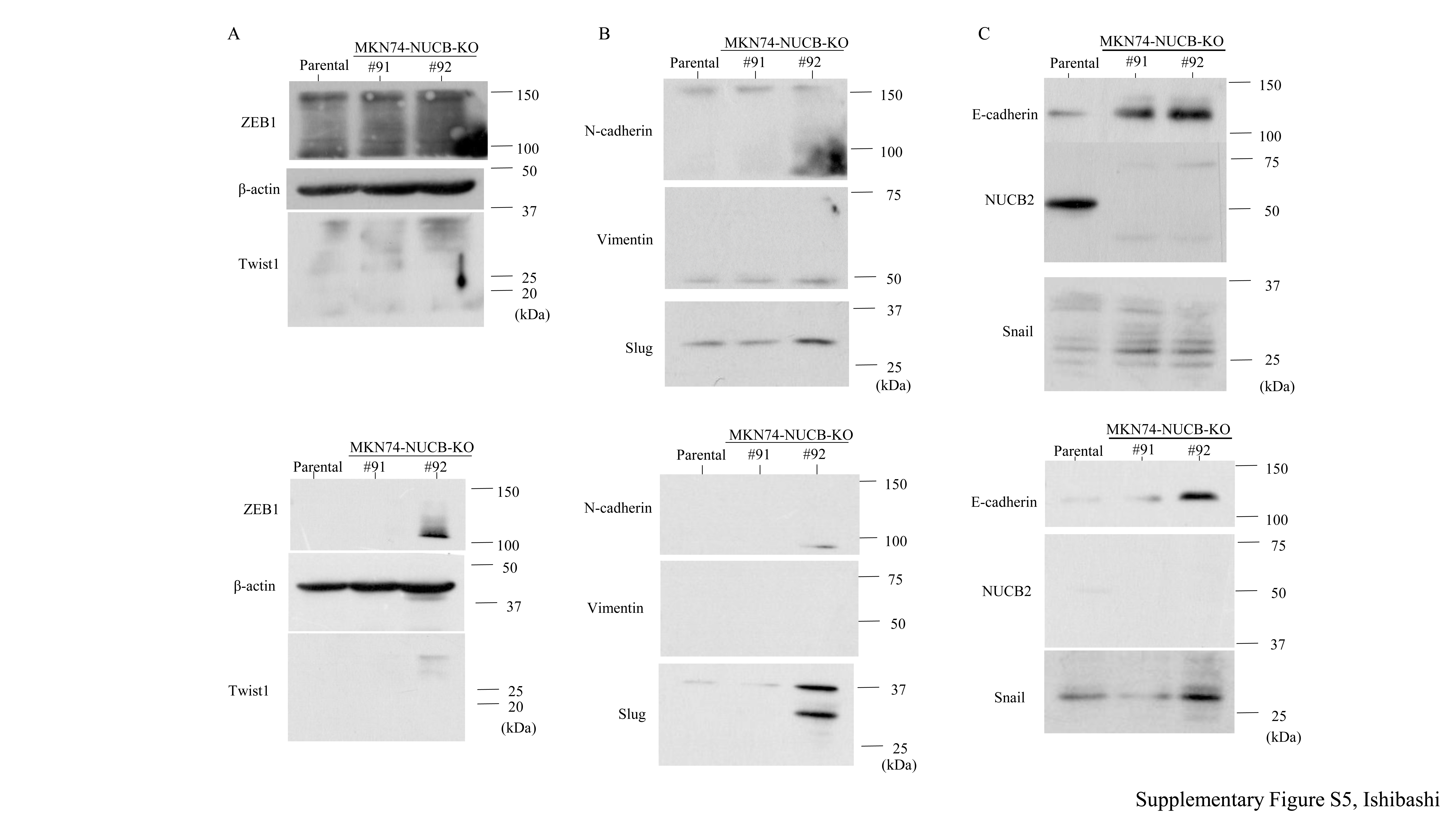

Supplement: Supplementary file 6 — Supplementary Figure S5. [file 41598_2024_61111_MOESM6_ESM.tif]
